# Technology for High-Sensitivity Analysis of Medical Diagnostic Images

**DOI:** 10.17691/stm2021.13.2.01

**Published:** 2021-04-30

**Authors:** S.R. Abulkhanov, O.V. Slesarev, Yu.S. Strelkov, I.M. Bayrikov

**Affiliations:** Associate Professor, Department of Engine Manufacturing Technologies, Samara National Research University, 34 Moskovskoye Shosse, Samara, 443086, Russia; Senior Researcher, Image Processing Systems Institute of the Russian Academy of Sciences (IPSI RAS) — Branch of the Federal Scientific Research Center “Crystallography and Photonics” of the Russian Academy of Sciences, 151 Molodogvardeyskaya St., Samara, 443001, Russia; Assistant, Department of Maxillofacial Surgery and Dentistry, Samara State Medical University, 89 Chapayevskaya St., Samara, 443099, Russia; Researcher, Video Data Mining Laboratory, Image Processing Systems Institute of the Russian Academy of Sciences (IPSI RAS) — Branch of the Federal Scientific Research Center “Crystallography and Photonics” of the Russian Academy of Sciences, 151 Molodogvardeyskaya St., Samara, 443001, Russia; Professor, Corresponding Member of the Russian Academy of Sciences, Head of the Department and Clinic of Maxillofacial Surgery and Dentistry, Samara State Medical University, 89 Chapayevskaya St., Samara, 443099, Russia

**Keywords:** radiodiagnosis, medical image, diagnostic image transformation, sensitivity of the transformed image to changes

## Abstract

Control and analysis of small, inaccessible to human vision changes in medical images make it possible to focus on diagnostically important radiological signs important for the correct diagnosis.

**The aim of the study** was to develop information technology facilitating the early diagnosis of diseases using medical images.

**Materials and Methods:**

To control changes in the image, we used its transformation based on solving a particular case of the knapsack problem. The proposed transformation is highly sensitive to any changes in the image and provides the possibility to record deviations visually with high accuracy. Medical images were obtained using cone beam computed tomography.

**Results:**

Practical evaluation of the information technology on tomograms showed the following: the transformed images of healthy bone tissue fragments from different parts of the jaw have similar shapes and nearly the same range of brightness. The transformed image of bone tissue after treatment has a shape close to that of the transformed image of healthy bone tissue. The transformed image of the affected bone tissue has a shape and brightness range differing from the shape and color of the transformed images of healthy bone tissue and bone tissue after treatment. However, transformation of medical images obtained with the Planmeca ProMax 3D Classic device (Finland) allows recording changes that account for less than 0.0001% of the entire image.

**Conclusion:**

The proposed method allows human vision to capture changes as small as nearly one pixel in the transformed image, which is impossible with the original medical image. Increasing the color contrast of the transformed medical image makes it possible to reveal the structure of the analyzed medical image fragment. The proposed image transformation method can be used for early diagnosis of diseases and in other fields of medicine.

## Introduction

Successful treatment of many diseases is determined by the stage at which the diagnosis is made. Early diagnosis allows timely identifying the disease cause and, as a result, administering more effective treatment.

Medical digital imaging with the use of radiologic technologies allows noninvasive visualization of the internal organs of the body for clinical analysis and medical intervention. However, radiologic technologies (fluoroscopy, radiography, X-ray computer diagnostics) cannot be used for early diagnosis due to the following circumstances:

a digital radiation detector does not allow determining the structure of changes in the area of interest of the medical image (MI);

the human organ of vision is unable to fix small changes in a static image [1, 2];

images obtained at different times will have systematic interference: it is impossible to ensure the same spatial position of the object under study at different times; the characteristics of the radiation source and its detector change over time.

The authors of studies [3–5] propose numerical modeling of biomechanical processes in medical practice based on models of continuum mechanics and numerical methods for solving the corresponding systems of differential equations. However, the proposed methods are unsuitable for early diagnosis of the disease for the following reasons:

the individual physical and technical parameters of the studied tissue of the patient’s body are unknown;

the individual characteristics of the metabolic processes in the studied biological tissue of the patient are unknown.

The use of statistical models [6–8] to identify and analyze the trends of small changes in the images of anatomical objects is unacceptable for the following reasons:

insufficient knowledge of processes occurring in biological tissues does not allow identifying all factors affecting the dynamics and nature of changes in the studied anatomical object;

clinical methods for diagnosing a disease call for a visual analysis of the image of the examined organ. Qualitative parameters (changes in the brightness of individual zones, the presence of neoplasms, etc.) useful for identifying the stage of the disease are subject to statistical processing. More complex MI parameters such as structure are inaccessible for perception by human vision, but it is these parameters that can be most significant for early diagnosis of the disease.

Identifying MI fragments that are most significant for diagnosing diseases is called segmentation [9–13]. Segmentation rules are formed using clinical methods for diagnosing diseases. However, modern clinical practice is unable to make an early diagnosis by detecting small changes in MI inaccessible for perception by the human visual system.

The methods under consideration suggest MI processing to be carried out based on a priori experience available to humans through the senses [1, 2]. However, the limited possibilities of human perception do not allow identifying all details of disease development.

We believe that MI should be transformed in such a way that small changes in the image are adapted for human perception. Transformed MI (TMI) visualization will enable medical personnel to make an early diagnosis without involving technicians.

Analysis of the literature in this area shows that the topic has been insufficiently explored.

The aim of the study was to develop information technology facilitating the early diagnosis of diseases using medical images.

## Materials and Methods

The transformed medical image should reveal small changes in the original MI that are inaccessible to human visual perception.

An acceptable method for transforming MI is described in [14]. The essence of the technique consists in solving a particular case of the problem of a two-dimensional knapsack [15–18], the size of which is bounded (the bounded knapsack problem).

To create TMI, we considered black and white MI. The weight of all items of a certain type corresponded to the number of pixels with a certain gradation of brightness — from white to black.

The number of items that we “placed in the knapsack” corresponded to the number of brightness gradations in the image, which could be a random natural number, including the most relevant numbers for MI: 256, 1024, and 16,384. We assumed the price of each item equaled 1. We carried out MI transformation on the basis of the theorem about the possibility of orthogonal arrangement of a sequence of non-overlapping squares whose total area is 1 in a square with an area equaling 2 [16–18]. We considered the number of pixels corresponding to a certain brightness gradation as the total number of MI^1^ pixels. Normalization of the pixel count for a particular brightness gradation allows abstracting from the image size. We interpreted each resulting ratio as the area of one square. The number of such squares is always finite since digital color palettes contain a finite number of colors. The sum of the areas of these squares is 1^2^. The side of each square is equal to the square root of the ratio of the pixel count with a particular brightness to the total number of MI pixels. We assigned a particular color of a certain color model (CM) to each MI brightness gradation from white to black [19]. We assigned the selected CM colors to the squares placed in the square with an area equaling 2. That was necessary to increase the contrast of human visual perception of TMI. Next, we arranged the squares in descending order of their areas. We propose to consider the arrangement of such a sequence of squares in the square with an area equaling 2 as the MI transformation.

**Figure 1:**
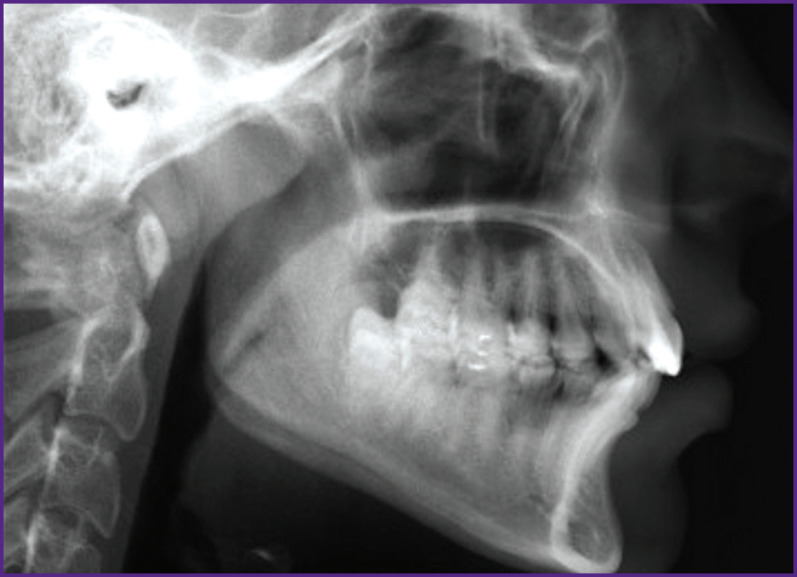
Tomogram of the human jaw

*For example, let us consider a tomogram of the jaw (Figure 1). The image is in .jpg format and consists of 945 rows and 1327 columns. Thus, this black and white image has a resolution of 945×1327=1,254,015 pixels.*

*A brightness gradation histogram^3^ representing the sequence of pixel count distribution for pixels of a particular brightness in the form of a table was created for this image in the MATLAB software environment.*

*Without losing the generality, let us show processing the image pixels, whose brightness gradation index from white to black equals 125. There are 5963 pixels in the image. Let us determine the proportion of these pixels in Figure 1:*

$$\frac{5963}{1,254,015}=0.004,755.$$

**Figure 2:**
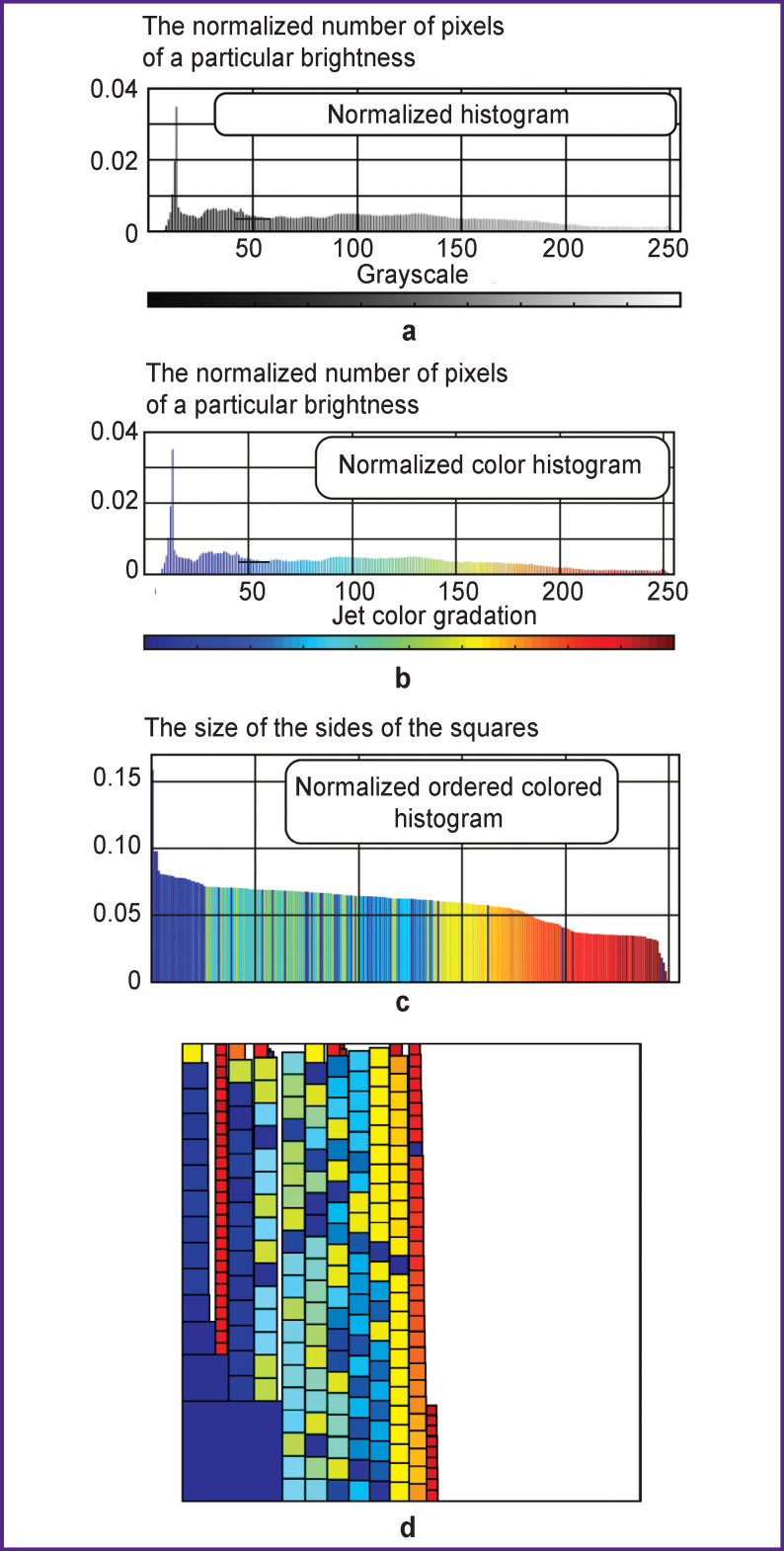
Transformation of histograms of the tomogram in Figure 1: (a) initial black and white histogram; (b) assignment of RGB CM colors to the values of the histogram in Figure 2 (a); (c) arrangement of the histogram in descending order by extracting the square root from the values of the histogram in Figure 2 (b); (d) the transformed medical image with RGB CM colors given to each square

*The resulting value is the normalized fraction of the number of pixels with a brightness index of 125 in the image (Figure 2 (a)).*

*Next, the number of pixels with brightness indices from 0 to 255 was determined in Figure 1. The number of such pixels was normalized, i.e. their fraction in the image was determined. The operation of normalizing the histogram is necessary to abstract from the MI size.*

*Then the normalized pixel counts of a certain brightness were arranged in descending order. This implies that the first element in a sequence of ordered normalized pixel counts was the largest normalized number of pixels of a particular brightness, while the last element was the smallest normalized number of pixels of a particular brightness.*

*The final operation was finding the square root of the normalized number of pixels of a particular brightness. For example, for a normalized pixel count with a brightness index of 125, this would equal* $\sqrt{0.004,755}=0.068,956,5.$

*In the proposed method, we interpret the value 0.068,956,5 as the side of the square.*

*We assigned a certain color of RGB CM (Figure 2 (b)) to each brightness gradation from white to black in Figure 2 (a). Figure 2 (c) shows the values of the histogram (Figure 2 (b)) arranged in descending order. In this case, we interpreted the values of the histogram in Figure 2 (b) as areas of squares. It means that the values of the histogram in Figure 2 (c) are equal to the square root of the corresponding values of the ordinates of the histogram in Figure 2 (b).*

*According to the works [16–18], we placed the sequence of squares with areas corresponding to the values of the ordered histogram in a square with an area of 2 (Figure 2 (d)). Giving the squares the colors of the selected CM (the histogram in Figure 2 (b)) increases the color contrast of TMI making it possible to reveal the structure of the analyzed MI. The selected RGB CM is best suited for the perception of colors by human vision_._*

To achieve our goal, we analyzed the biotransformation dynamics of osteoplastic material in the recipient bed in the postoperative period when the patient was under medical supervision.

Cone beam computed tomography (CBCT) and lateral teleradiography of the head were used as methods for visualizing the biotransformation dynamics of the graft. A Planmeca ProMax 3D Classic device (Planmeca, Finland) was used to perform CBCT following the standard requirements for the imaging of the subject under study. The imaging mode “M” (adult) was selected with an X-ray tube voltage of 90 kV and current of 6.3 mA; volume diameter — 50 mm, volume height — 80 mm, dose area product (DAP) — 472 mGy·cm^2^; computed tomography dose index (CTDY) — 4.6 mGy. The tomogram was a black and white gray-level bitmap image with a brightness depth of 8 bits.

To monitor the dynamics of the disease, it is necessary to transform not the whole MI, but only those fragments (zones of interest) that the doctor considers the most significant for diagnosis. The shape of such a fragment and its position on the tomogram should be determined by the doctor relying on his practical expertise. Let us consider applying the proposed method to a specific case.

**Figure 3:**
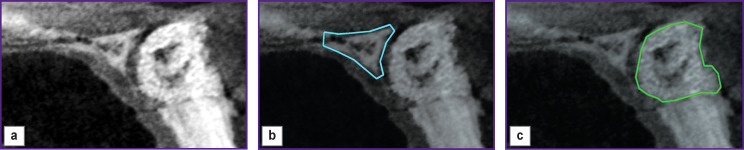
Tomogram of the patient’s jaw joint performed during the first visit: (a) original image; (b) the zone of healthy bone tissue marked out by the doctor; (c) the area of the affected bone tissue marked out by the doctor

*Patient Kh. (the case history was described in [19]) was reported to have an odontogenic cyst. To replace the affected bone tissue, we used osteoconductive granular bone graft material of animal origin. The same doctor carried out affected management and treatment. Figure 3 shows the tomogram performed during the first visit of Kh. to the doctor.*

*Figure 3 (a) shows a tomogram of the jaw joint with the affected bone tissue. In Figure 3 (b), (c) there are fragments of healthy and affected bone tissue of the same joint. The fragments of healthy and diseased bone tissue images were marked out by the attending physician. Table 1 shows the dimensions of the image and selected fragments.*

**Table 1:** The sizes of images and selected fragments in Figure 3

Images	Figure 3 (a) — tissue at the time of the first visit to the doctor	Figure 3 (b) — healthy bone tissue	Figure 3 (c) — affected bone tissue
Image size (pixels)	709×1174=832,366	591×936=553,176	591×941=556,131
The number of pixels in the selected fragment	—	30,129	75,454

*The treatment involved removing the affected bone tissue, which was replaced with granular material from bone tissue of animal origin (xenograft). Over time, the xenograft was integrated into the bone tissue of the lower jaw.*

**Figure 4:**
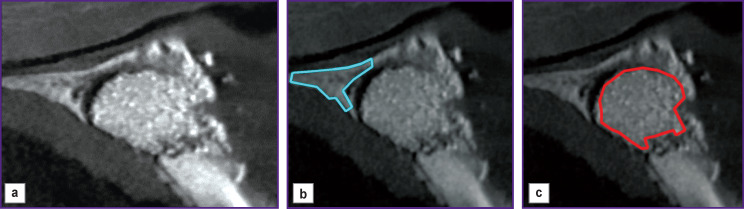
The patient’s tomogram recorded after treatment: (a) the image at the time of the last call; (b) the zone of healthy bone tissue marked out by the doctor; (c) the area of regenerated bone tissue marked out by the doctor

*Figure 4 (a) shows a tomogram of bone tissue with the implanted xenograft. In Figure 4 (b), (c) there are fragments of healthy and regenerated bone tissue of the same joint, marked out by the attending physician. Table 2 shows the dimensions of the image and selected fragments.*

**Table 2:** The sizes of images and selected fragments in Figure 4

Images	Figure 4 (a) — tissue at the time of the last visit to the doctor	Figure 4 (b) — healthy bone tissue	Figure 4 (c) — regenerated bone tissue
Image size (pixels)	1709×1875=3,204,375	1063×1053=1,119,339	1563×1753=2,739,939
The number of pixels in the selected fragment	—	36,147	109,255

*Tomograms in Figures 3 and 4 were performed with a timing difference of 3 months. We assumed that in the operating conditions of the dental clinic, there is practically no burnout of the X-ray apparatus filament during this time in accordance with the data [20, 21]. For this reason, we performed no brightness correction for the tomograms in Figures 3 and 4.*

**Figure 5:**
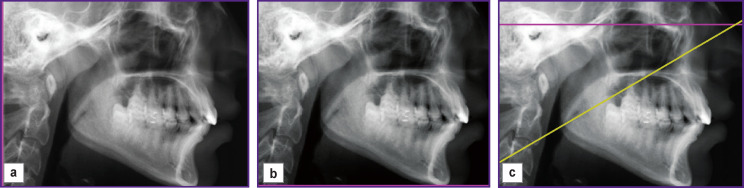
The tomogram in Figure 1 with data loss: (a) tomogram without the 1^st^ column; (b) tomogram without the last row; (c) tomogram without the 47^th^ row and without an oblique segment

*Image coordinate axes in Figures 3 and 4 differ as a result of a change in the spatial orientation of the patient’s lower jaw relative to the X-ray source. This circumstance leads to brightness changes on tomograms made at different times. To diagnose the early stage of the disease by MI, it is necessary to superimpose such images. Therefore, we displaced the image in Figure 4, rotated it by certain angles, and scaled it. The values of displacement, angles, and scaling coefficient were determined using reference points [22–26]. The reference points on the image (see Figure 3 (b), (c)) coincided with the vertices of the polygons of interest: (b) a fragment of healthy bone tissue selected by the doctor; (c) a fragment of the affected bone tissue. There were at least 14 stereo pair points (see Figure 4 (b)). The coordinates of the points in Figure 5 (b), (c) corresponding to the reference points in Figure 4 (b), (c) were determined using the bundle method of phototriangulation [27, 28].*

We investigated the dynamics of graft biotransformation using the proposed method. For that purpose, we carried out a comparative analysis of the tomograms of the jaws before and after filling the bone defect in two groups of patients: group 1 (n=10) was the test group, group 2 (n=20) — the control. Bone defects were filled with two types of biodegradable granular osteoplastic material. In the test group, we used a multicomponent augmentate, in the control group — osteoconductive xenogenic material. The study complies with the Declaration of Helsinki (2013) and was performed following approval by the Ethics Committee of the Samara State Medical University. Written informed consent was obtained from every patient.

**Figure 6:**
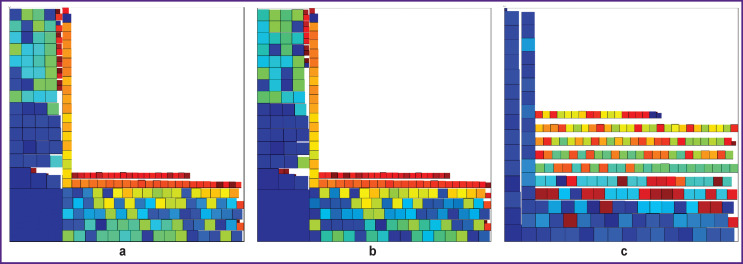
Transformed medical images: (a) Figure 5 (a); (b) Figure 5 (b); (c) Figure 5 (c). Image dimensions — 450×450

**Figure 7:**
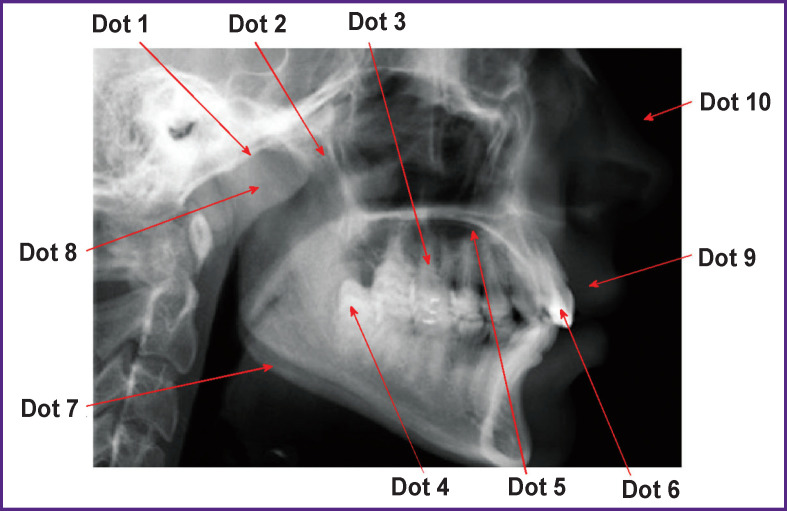
Randomly selected dots on the tomogram image

The quality of bone tissue restoration was studied 6 months after filling the bone defect: in patient S., 29 years old, the test group (Figure 6); in patient A., 33 years old, the control group (Figure 7).

Figure 6 (b) shows the result of selecting a fragment of healthy bone tissue adjacent to the defect to obtain an individual control indicator of the norm. Figure 6 (c) shows a fragment of remolded multicomponent material in a bone defect.

## Results

Sensitivity of the proposed method of MI transformation to changes in the image has been studied. To do this, we removed one column, one row, and at the same time one row and an oblique segment (highlighted in different colors) in the bitmap image of the lower jaw in Figure 5. In all three cases, in Figure 6 there was a loss of image data shown in Figure 1 (945×1327 pixels):

loss of one column in Figure 6 (a) — 945/ (945×1327) · 100% =0.075%;

loss of one row in Figure 6 (b) — 1327/ (945×1327) · 100%=0.11%;

loss of a row and oblique line in Figure 6 (c) — 2 · 1327/(945×1327) · 100% =0.22%.

Changes in Figure 5 (a)–(c) are well visible in Figure 6.

**Figure 8:**
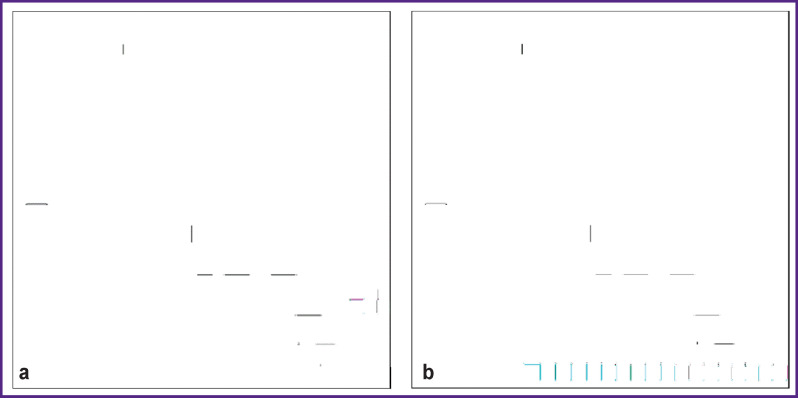
Images obtained by subtracting the transformed tomogram image without one dot (pixel) from the original transformed image in Figure 1: (a) dot 6; (b) dot 3. Image dimensions — 450×450

In the image (see Figure 1), we deleted 10 dots randomly and sequentially (see Figure 7). The loss of each dot (pixel) of the image resulted in data loss of less than 0.0001% — 1/(945×1327)∙100%=0.000,07%. Analysis of the image with the lost dot showed that it was impossible to fix the loss of one pixel visually. We subtracted the transformed image in Figure   1 without one pixel from the transformed original image in Figure 2. Additionally, we increased the contrast in the image resulting from subtraction by 10 times. Figure 8 shows the images with the smallest and largest differences between the original image and those with a lost pixel.

It can be seen in Figure 8 that the loss of one pixel changes the structure of the entire transformed image. This circumstance meets the requirement that should be observed when transforming the diagnostic image: small changes in the original image inaccessible for human visual perception should determine the picture in the transformed image.

**Figure 9:**
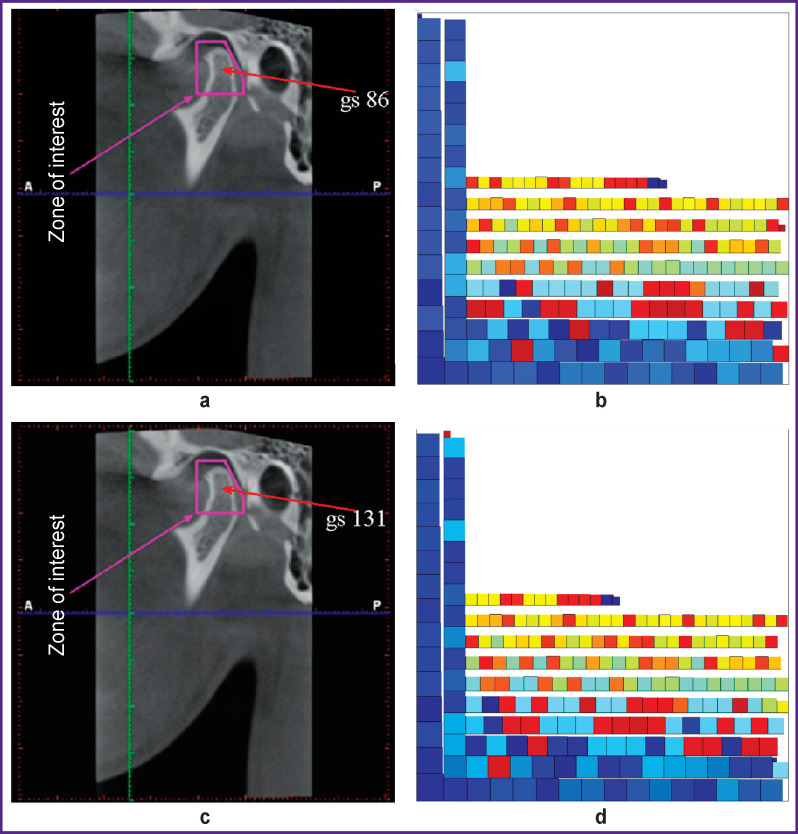
Changing the pixel brightness in the tomogram zones of interest: (a) an image of the zone of interest with indication of a pixel with brightness of 86; (b) a transformed image of the zone of interest in Figure 10 (a); (c) an image of the zone of interest with indication of a pixel with brightness of 131; (d) a transformed image of the zone of interest in Figure 10 (b)

**Figure 10:**
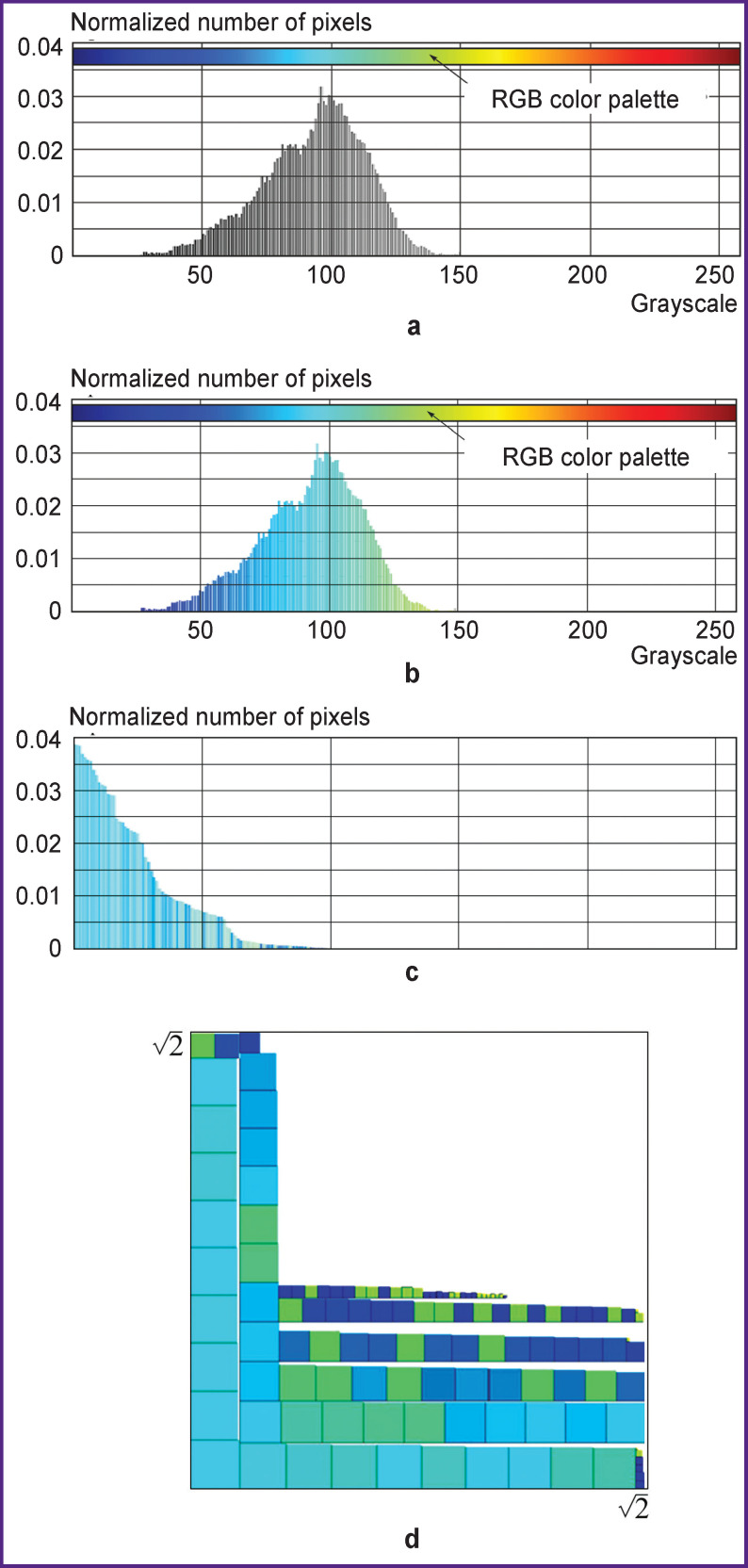
The sequence of transforming the zone of interest in Figure 4 (c): (a) a histogram of a black and white image; (b) assignment of RGB CM colors to the histogram of the zone of interest; (c) histogram of the zone of interest arranged in descending order; (d) a transformed image of the zone of interest in Figure 3 (b) (healthy bone tissue)

We changed the MI and did not remove a pixel, but changed its brightness (Figures 9, 10). Figure 9 shows a tomogram with the zone of interest highlighted by the doctor. In this zone, we have selected a pixel with brightness of 86. In the same image, we assigned (gave) the selected pixel brightness value of 131. Both images were transformed (Figure 9 (b), (d)). The obtained figures show that a change in the brightness of one pixel leads to a change in the transformed image of the zone of interest.

**Figure 11:**
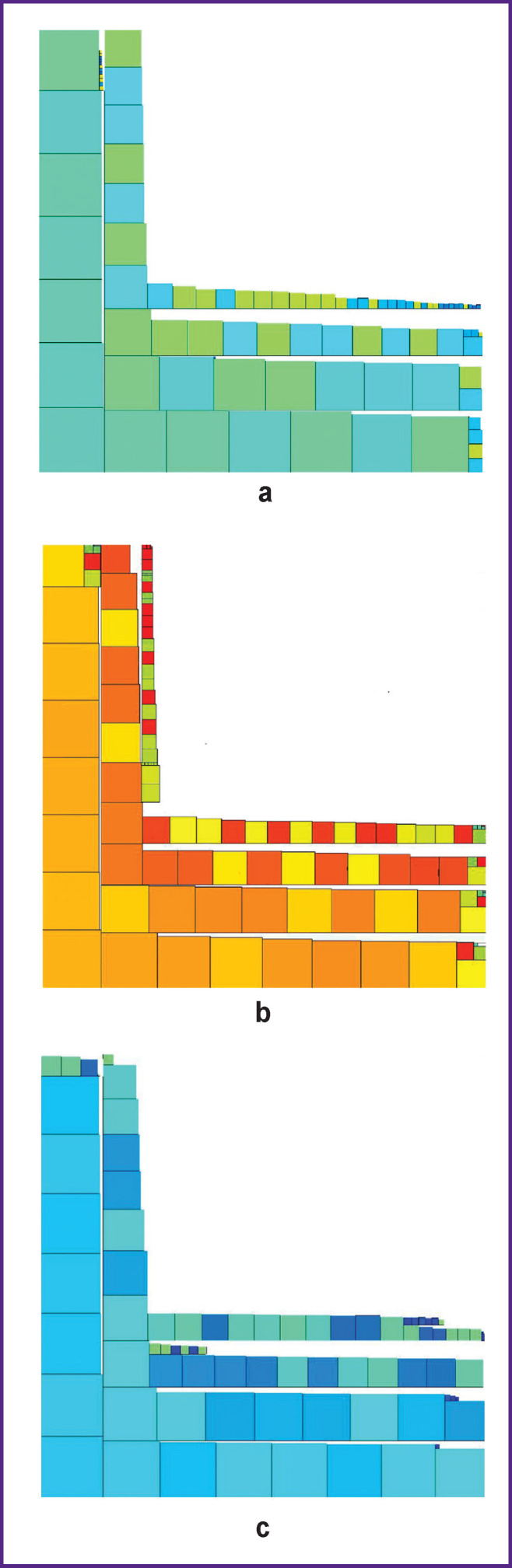
Transformed images of various bone tissue fragments: (a) healthy bone tissue (Figure 4 (b)); (b) affected bone tissue (Figure 3 (c)); (c) bone tissue after treatment (Figure 4 (c))

Figure 10 shows the sequence of transforming the zone of interest in Figure 3 (b) (healthy bone tissue). We used it to transform the zones of interest in Figure 3 (c) and Figure 4 (b), (c) (Figure 11). Figure 11 clearly shows the differences between healthy, affected, and regenerated bone tissue, while the figures in the transformed images in Figure 10 (c) and Figure 11 (a) have almost similar^4^ shapes and approximately the same range of brightness. This circumstance is explained by the fact that both images correspond to healthy bone tissue. The difference between Figure 10 (c) and Figure 11 (a) is explained by the observation time: the interval between images was 3 months. During this time, the patient’s condition could have changed for various reasons. Therefore, it becomes necessary to create a structural criterion for the physiological norm of the tissue under study.

The patterns in the transformed images (Figure 11 (a), (c)) have similar shapes but differ in the range of brightness. This is determined by the fact that the bone formed after treatment is close to the healthy tissue in structure, but it also has differences.

The results of using the proposed method for visualizing the dynamics of changes in tomograms of the jaw with a bone defect showed the following possibilities for disease diagnosis:

making an early diagnosis;

identifying visually with high accuracy the patterns characteristic of different stages of the disease;

providing a high degree of diagnosis objectivity.

Visually, the difference between two images can be detected with an accuracy of 1 pixel, although we consider this accuracy to be excessive.

It should be noted that the use of the proposed method requires a bitmap MI. It is no matter what physical methods are used to obtain the image. It can be X-ray, computed tomography, MRI, and other methods where bitmap images are used for visualization.

## Discussion

The proposed MI transformation method allows visualizing changes in the image up to one pixel, which can be less than 0.0001% of the image. We do not know other visualization methods providing the possibility to record changes in the controlled image with such accuracy. Visualization of small changes is achieved by transforming the image. The result of the transformation is the adaptation of small changes to human visual perception that has natural limitations in resolution.

Transformation uses information from the entire diagnostic image, and not only the part of it that has undergone changes resulting from the disease. For this reason, early diagnosis based on the transformed image will provide the most reliable diagnosis of the disease.

Application of CMs focused on the perception of colors by human vision makes it possible to reveal the structure of the analyzed zone of interest in the MI while forming a transformed image.

The proposed method is applicable for transforming MI regardless of the physical processes through which they were obtained. The only condition is the following requirement: the image must be bitmap and grayscale, for example, black and white. In case it is observed, the proposed MI transformation method can become universal for the early diagnosis of various diseases.

The sensitivity of the method can lead to recording the changes in the characteristics of technical means (sensors and radiation sources) in MI, which will hinder early diagnosis. Besides, we believe that the change in the brightness of small groups of pixels in the image can occur within the physiological range. For these reasons, the problem of possible changes in MI within the physiological range remains unsolved.

Analysis of the diagnostic method of image transformation makes it possible to formulate the following directions for future research.

1. Performing a numerical parameterization of the transformed image to study the dynamics of the disease course.
2. Developing a technique for objectively identifying the zone of interest based on particular criteria, since the zone of interest is determined on MI by the attending physician based on their clinical experience and therefore the subjective factor influences image transformation results, reducing reliability of the disease diagnosis.
3. Creating a special filter to compensate for changes in the characteristics of technical means and MI changes within the physiological range.

In addition, a special case of solving the knapsack problem was used when forming the transformed image. In doing this, we assumed that the price of each item placed in the knapsack was equal to 1. This reduces the versatility of the MI transformation method. Therefore, the price of each item can be interpreted as individual characteristics of the patient (for example, intolerance to certain drugs).

Solving these problems will allow making an early diagnosis and improving its objectivity.

## Conclusion

There has been developed a high-sensitivity technology for analyzing diagnostic images that provides the possibility to record changes on tomograms inaccessible to human vision with a high degree of objectivity, accuracy, and reliability.

The proposed method for visualizing small changes in the transformed medical images is suitable for use in telemedicine. Transformation of medical images provides additional opportunities for their processing by other methods, for example, using convolutional neural networks [29–33]. Moreover, the structure of the transformed medical image expands the functionality of various sensors and probes in the analyzed image coordinate system [34–38].

## Footnotes

1 This transformation of pixel counts of different brightness in the image is called normalization. The normalized number of pixels of a particular brightness has no dimensions and varies in the range from 0 to 1.

2 The sum of pixel counts corresponding to different brightness gradations equals the total number of pixels in the image. The ratio of the sums of all these groups of pixels to the total number of pixels in the image is 1.

3 Histogram is a bar graph where the ordinate of each bar corresponds to the number of pixels of a particular brightness gradation on the analyzed image, the abscissa corresponds to the index of a particular brightness gradation.

4 Similar shapes mean squares shaped similarly to those placed in the square of area 2.
